# Editorial: Reproductive health and mental health in LMICs: adolescent health

**DOI:** 10.3389/frph.2024.1383170

**Published:** 2024-03-01

**Authors:** Margaret I. Fitch

**Affiliations:** Bloomberg Faculty of Nursing, University of Toronto, Toronto, ON, Canada

**Keywords:** adolescence, adolescent sexual health, adolescent well-being, adolescent mental health, adolescent reproductive health

**Editorial on the Research Topic** Reproductive health and mental health in LMICs: adolescent health

For adolescents, the interaction between reproductive/sexual health and mental health is a critically important consideration for their overall health and well-being. Each topic area is of significance in itself, but evidence is emerging that there is a clear association between the two domains with long-term implications for the individual and for wider society. This is a topic area that requires increased awareness and attention to tailored service provision.

Mental health disorders are common on a global scale (1), contributing as much to disability and shortened life spans as cardiovascular disease (2). One-quarter of the world's population are between 10 and 24 years of age with as many as 20% of these adolescents possibly experiencing a mental health problem in any given year (3). Fifty percent of these disorders begin by the age of 14 years and 75% by 24 years (4). Unfortunately, 75% of the young people with mental health problems are not receiving the help they need (5). Additionally, recent reports indicate these rates are increasing year by year (6).

Eighty-six percent of the global population of adolescents are living in low-and middle-income countries (4). It is predicted that Africa alone will contain 37% of all people under the age of 18 years by 2050 (7). These young people are the future of our societies, and their health and decision-making will have implications for their own well-being, and that of their families and communities. It is imperative health care systems respond to the critical need for support and treatment of mental health disorders in adolescents.

The features and the profiles of mental health disorders are broadly similar between high-income and low- and middle-income countries, but the availability of resources means that the gaps in treatment are much larger in the latter settings (8). The leading mental health disorders experienced by adolescents are anxiety and depression, both of which have effective interventions (4). Overall, more young people are experiencing mental health issues today than 30 years ago. For example, rates of depression in adolescents increased from 8.1% in 2009 to 15.8% in 2019 in the USA (9) and 11.5% experiencing clinically significant or severe depression in 2023 (10). Data from low- and middle-income countries are more difficult to obtain, but available evidence points to indications of even higher rates in these settings (11). For example, rates are reported as 17% for South Africa (12) and between 14.8% and 22.7% in Taiwan (13). Recent reports indicate that rates increased significantly during the worldwide pandemic of COVID-19 (14).

A similar picture exists for the disorders of anxiety. When the disorders of excessive uneasiness, worry and fear; generalized anxiety; obsessive-compulsive behaviors; and post-traumatic stress are combined, they account for a prevalence of 32% in 13- to 18-year-olds (6). In high-income settings, the prevalence of anxiety disorders (including specific phobias) over a 12-month period was reported as between 7.1% and 18.4% (15), while a systematic review of mental health disorders in adolescents during the pandemic reported a prevalence for anxiety at 43.69% in low- and middle-income settings (16). Overall, clinically elevated anxiety symptoms (1 in 5 adolescents) doubled during the first year of the pandemic (17).

Adolescence is a time of transition where profound physical, emotional, psychological, and social changes are occurring. It is the phase of life between childhood and adulthood, marked by the onset of puberty and heralding a unique stage of human development. Thus, it is a critical time for laying the foundation for good health. Importantly, it is a time when the young person is coming to terms with self-identify, independence and autonomy, and is also a critical time for developing social and emotional habits. Roughly the period corresponds to the years of 10–19. However, there are various age ranges used in reporting on this population, making comparisons across settings somewhat challenging.

The mental health of adolescents is affected by the social determinants of health. These include in particular poverty, food security/nutrition, neighborhood/community, trauma, and racism (18). The list of potential risk factors for experiencing mental health problems in adolescence is lengthy and includes loss or death of a loved one, moving homes, divorce of parents, changing schools, traumatic events, having a long-term physical illness, lack of peers, or birth of a new sibling (19). Added to these types of life events, adolescents today are living in a rapidly changing world with many global issues confronting all of us. Uncertainty about the future is a predominant concern for much of the world's population and can add additional burden to the challenge of achieving and maintaining mental health.

There are unique disadvantages and vulnerabilities for adolescents as they manage the changes brought about by their age and changing bodies. Reproductive/sexual health is one challenge that is particularly subject to the vulnerabilities of youth (20). There are unique challenges and consequences for them as they navigate the reproduction/sexual health issues and their related behaviors.

Reproductive/sexual health addresses the processes, functions and system at all stages of life of producing offspring. It encompasses the rights of both men and women and deals with more than physical matters. It includes a person's right to a healthy body; to the autonomy, education, and healthcare that allows free decision-making about who to have sex with; to have a safe and satisfying sex life; to have the capability to reproduce and the knowledge about how to avoid sexually transmitted disease and unwanted pregnancy. It is an important and integral part of overall health and well-being. Having the autonomy to make decisions about sexuality and reproduction is seen as a human right. It is grounded in the rights to health, to be free of sexual violence, to decide to become pregnant, and to be informed.

For adolescents, the reproductive/sexual challenges are many (11, 21). Commonly, adolescents are expected to negotiate puberty, come to terms with their bodily changes, gain a clear sense of personal and sexual identify, develop new cognitive skills such as abstract thinking, and learn more about managing emotional responses. Generally, adolescents want to stay free of unwanted pregnancy, unsafe abortion, sexually transmitted diseases (including HIV/AIDS), and all forms of sexual violence and coercion. Girls especially face the potential for high mortality rates associated with pregnancy, unsafe abortion practices, early and forced marriages, early and unintended childbearing, trafficking, intimate partner violence, and exploitation.

Recent reports indicate these issues remain of global concern (20). For example, sixteen million girls aged 15–19 give birth annually, 95% of which are in low-middle-income settings. This age group faces higher risks of complications from pregnancy than older women and are left with subsequent challenges in terms of education and supporting a child. Worldwide, primarily because of unintended pregnancy, 4.5 million adolescents undergo abortion, of which 40% are deemed unsafe. Additionally, adolescents are most severely impacted by HIV/AIDS. Those between 15 and 24 years account for 41% of all new HIV infections and it is estimated there are 5 million of these young people living with the disease. Many are girls who frequently are not aware of their HIV status.

Certain challenges related to reproductive and sexual health which adolescents face vary from country to country influenced by culture, religion, and economics (4). These challenges have been documented as lack of knowledge and ability to access information about reproductive/sexual health, early marriage and pregnancy, limited access to contraceptives/condoms and safe abortions, and girls limited power over their sex lives (22). Engaging in risky behaviors (e.g., alcohol use, drug use), stigma, and evolving decision-making skills can increase the degree of potential for unwanted consequences. Additionally, not all adolescents are affected equally. Orphans, young girls living in rural settings, individuals experiencing physical or mental impairments, or those in abusive situations are at higher risk.

Mental health is a key aspect of achieving good reproductive/sexual health. An increasing amount of research has emphasized a bi-directional relationship between the two variables and the complex, dynamic and multifaceted nature of that interaction. Numerous social, cultural and biological factors can influence the outcomes. The issues of reproductive/sexual health and mental health have been characterised as two sides of the same coin. The prevalence of depression during pregnancy for adolescents was reported as ranging widely, from 2.0% to 89.1% across 28 studies from various countries around the globe (23). The prevalence for anxiety ranged from 13.6% to 19.2% while stress was cited as 22.5% to 40.5%. Suicidal ideation ranged from 4.2% to 8.9% while suicide range from <0.1% to 13.3%. During the postpartum period depression ranged from 2.5% to 57%. The prevalence of depression following abortion for adolescent girls was reported as 16.1% to 85.0%. Most studies report higher rates of mental health disorders in adolescents related to pregnancy that for older women.

Advocates are calling for increased recognition of adolescents' challenges and service development tailored to the unique needs of adolescents. In many countries, adolescent population health is not seen as a priority and there is a lack of adolescent friendly services which integrate the two topic areas (24). Gaps exist in availability and easy access to comprehensive information about reproductive/sexual health and about mental health. Availability and access challenges also exist to relevant services with professionals trained in caring for adolescents and assisting with these topic areas in truly supportive ways. Specifically, access to information about contraception, availability of contraceptives and condoms, and access to prenatal care for teens, safe abortion and abortion after-care are sadly lacking in many settings. Addressing these challenges requires action in many directions—research, education, health practices, policy—and collaboration between sectors (25). Health is foundational to a country's economy and societal development. Adolescents are a country's future.

As action is taken to implement adolescent friendly services, the foundation must begin with understanding the unique needs of the population. However, recognition must be given to the reality this population is not homogeneous. We must understand the variations in the population and the unique perspectives and concerns of groups with different characteristics or living in different circumstances. Qualitative research can offer unique data to contribute to this aim and begin to acknowledge the intersectoral nature and complexity of their life experience conditions. The three papers in this Research Topic do just that. Each offers a perspective from a unique sector of the adolescent population—adolescents from South Africa living with HIV Bergam et al., young men living in low-and middle-income countries Mhlongo et al., and adolescent immigrants to Canada Meherali et al. These papers provide good examples of the type of research needed to enlarge our understanding of the intersection between reproductive/sexual health and mental health for adolescents.
